# Postoperative Margin Management After Conventional Excision of Facial Basal and Squamous Cell Carcinomas: A Histotype‐Specific Framework

**DOI:** 10.1155/jskc/9952281

**Published:** 2026-08-28

**Authors:** Flavio Perottino, Stefano Ottoboni, Giulia Dalmasso, Katia Barbaro, Daniele Sagrafoli, Paola Ghisellini, Cristina Rando, Roberto Eggenhöffner, Mario Borgione

**Affiliations:** ^1^ Department of ENT and Stomatology, Centre Hospitalier des Escartons, Briancon, France; ^2^ Department of Surgical Sciences and Integrated Diagnostics (DISC), University of Genoa, Genoa, Italy, unige.it; ^3^ Experimental Zooprophylactic Institute of Lazio and Tuscany, Rome, Italy

**Keywords:** basal cell carcinoma, conventional excision, facial reconstruction, non-melanoma skin cancer, postoperative surveillance, recurrence, skin cancer, squamous cell carcinoma, surgical margins

## Abstract

**Background:**

Postoperative management of close or positive margins after conventional excision of facial nonmelanoma skin cancer is challenging, especially in elderly patients and anatomically constrained sites where re‐excision may impose functional or aesthetic morbidity.

**Methods:**

We retrospectively analysed 238 facial carcinoma cases treated by conventional excision at a peripheral ENT unit, including 172 basal cell carcinomas (BCC) and 66 squamous cell carcinomas (SCC). Margins were classified as clear, close or positive. Outcomes were analysed descriptively; two‐sided Fisher exact tests were used as exploratory descriptors of distributional imbalance under sparse‐event conditions, not as tests of treatment superiority or equivalence.

**Results:**

In BCC, no recurrence occurred after clear margins (0/90). Among non‐clear‐margin BCCs, recurrence remained uncommon after structured surveillance (5/65) and early re‐excision (1/17). In SCC, recurrence occurred only after positive margins (3/15), with none after clear or close margins.

**Conclusion:**

The findings support histotype‐specific postoperative interpretation of margin status after conventional facial skin cancer excision. In this cohort, selected lower‐risk BCC cases were managed through structured surveillance, whereas SCC recurrence was confined to positive‐margin cases, supporting a lower threshold for timely re‐excision or equivalent oncologic management when clinically feasible.

## 1. Introduction

Basal cell carcinoma (BCC) and squamous cell carcinoma (SCC) together account for the overwhelming majority of facial nonmelanoma skin cancer (NMSC), and their surgical management occupies a substantial portion of ENT and facial surgery caseload in elderly populations [1–3]. Both tumours are primarily treated by surgical excision, and both can be cured by complete removal. BCC and SCC differ in their patterns of subclinical extension, their potential for nodal spread and perineural invasion, and—most relevant to this paper—in what a close or positive postoperative margin actually means in clinical practice [2, 4, 5].

A positive margin does not carry the same prognostic and management implications in BCC and SCC. Residual BCC usually grows slowly, remains predominantly local and may not become clinically evident during short‐ or medium‐term follow‐up, particularly in elderly patients. Residual SCC carries a meaningfully higher risk of progression, deeper extension and—in the worst cases—nodal or distant spread [4–6]. A uniform postoperative algorithm applied indiscriminately to both histotypes is not biologically justified, because the balance between residual‐disease risk and re‐excision morbidity differs substantially between BCC and SCC.

Facial anatomy further modifies this decision with its own anatomical constraints. Subunits such as the nasal ala, the medial canthus, the auricular concha and the periauricular sulcus offer little expendable tissue, and surgical re‐excision in these areas may carry substantial functional and aesthetic morbidity: eyelid ectropion, nasal airway compromise, auricular deformity or consumption of skin capital that an elderly patient with field cancerisation will need for future lesions [7–9]. The decision to re‐excise or observe is therefore not purely oncological. It is a clinical judgement that must weigh biological risk against surgical morbidity, patient frailty and the feasibility of long‐term follow‐up.

Mohs micrographic surgery offers margin‐controlled excision and is the preferred approach for high‐risk facial NMSC when available [10–12]. Its availability, however, is not universal. Many peripheral and regional centres, including the study institution, rely on conventional excision as standard practice. In this real‐world context, postoperative margin management must be guided by histopathological assessment, clinical judgement and a structured surveillance protocol.

Conventional excision remains clinically important for skin cancer care because access to Mohs surgery and other margin‐controlled techniques is uneven across health systems. In this setting, the postoperative question is not whether conventional excision can replace Mohs surgery in high‐risk cases, but how clinicians should interpret close and positive margins when conventional excision is the actual treatment pathway available to the patient. This question is particularly relevant for elderly facial skin cancer patients, in whom residual‐disease risk, reconstructive morbidity, functional preservation and realistic follow‐up capacity must be considered together.

The literature on postoperative management of nonclear margins after conventional excision remains limited. Most published series focus on recurrence rates after Mohs surgery, or on incomplete excision rates after standard excision, without specifically addressing the re‐excision versus surveillance decision in the context of anatomically constrained facial NMSC in elderly patients [13–17]. This study aims to address that gap.

We report a single‐centre retrospective cohort of 238 consecutive facial NMSC cases managed by conventional excision over a 10‐year period. The aims are threefold: to describe how histotype, margin status, anatomical site, patient vulnerability and reconstructive feasibility jointly shaped postoperative decisions in real‐world clinical practice; to examine whether observed recurrence distributions were clinically coherent with distinct BCC and SCC biology and to propose a pragmatic, histotype‐specific decision framework for skin cancer margin management that remains calibrated to the features of this retrospective descriptive dataset.

## 2. Materials and Methods

### 2.1. Study Design and Setting

This was a retrospective observational study of facial BCC and SCC managed by conventional surgical excision at the Centre Hospitalier des Escartons, Briancon, France, between January 2014 and December 2023. Because lower‐risk lesions were treated in the ambulatory setting, the cohort consisted predominantly of higher‐risk facial tumours managed in the operating theatre. The institutional setting is clinically important to the interpretation of this paper: Mohs micrographic surgery was not routinely available, and postoperative management decisions were therefore made entirely based on conventional histopathological margin assessment, clinical review and the surgical team’s judgement. No written institutional protocol governed the choice between surveillance and early re‐excision; decisions were made by the operating surgeon on a case‐by‐case basis, guided by published evidence, guideline concepts and clinical experience. Management comparisons are therefore nonrandomised. Anatomical risk was categorised according to the Italian Association of Medical Oncology (AIOM) site classification: high‐risk sites comprised the eyelids, eyebrows, periorbital region, nose, lips, chin, pre‐ and post‐auricular regions, temples and ears, with the cheeks and forehead treated as intermediate‐risk. This classification underlies the high‐risk and anatomically constrained groupings used throughout.

All specimens were oriented using sutures or anatomical landmarks, submitted for routine histopathological assessment, inked to identify surgical surfaces and processed by standard vertical bread‐loaf sectioning. Pathology reports provided tumour histotype, histological subtype when available, margin status and depth descriptors. Because retrospective pathology reports did not allow systematic stratification of margin topography (deep versus peripheral, focal versus extensive) across all cases, these variables were considered clinically during decision‐making but were not used for subgroup analyses. Histological subtype data are provided in Supporting Tables S-II and S-III.

### 2.2. Case Selection and Unit of Analysis

The study included 238 histologically confirmed facial NMSC cases treated by conventional excision, comprising 172 BCC and 66 SCC cases. Eligible cases were adult patients with facial BCC or SCC who underwent surgical excision and had postoperative follow‐up of at least 12 months, or a documented oncological event before 12 months. Cases managed exclusively with nonsurgical treatment, including radiotherapy or topical therapy, and cases presenting with metastatic disease were excluded. Each included case corresponded to a surgically treated facial carcinoma followed for postoperative outcomes. Local recurrence was attributed to the treated lesion when carcinoma arose on, or within 1 cm of, the surgical scar. Clinically distinct new carcinomas arising outside this area were recorded as second primary tumours and were not counted as local recurrence of the index lesion. All denominators therefore refer to treated carcinoma cases unless otherwise specified.

### 2.3. Margin Classification

Margin status was classified using explicit ink‐based operational definitions applied consistently across the cohort. A clear margin was defined as tumour separated from the inked surface by at least 1 mm of uninvolved tissue. A close margin was defined as tumour less than 1 mm from the inked surface but not reaching ink. A positive margin was defined as tumour at the inked surface—tumour on ink. These definitions were applied uniformly to peripheral and deep margins. The 1‐mm threshold defining a close margin remained unchanged throughout the study period (2014–2023), so that margin categories are directly comparable across the cohort.

### 2.4. Reconstructive Approach

Reconstruction was selected at the time of primary excision according to defect geometry, tissue laxity, exposure of deep structures, facial subunit and functional requirements. Closure modalities included primary closure, local flap reconstruction, full‐thickness skin grafting, combined flap and graft reconstruction and second‐intention healing. Reconstructive modality was analysed as a procedural marker of anatomical and operative complexity, not as an independent oncological variable.

### 2.5. Follow‐Up Protocol

SCC cases were reviewed every 3 months in the first postoperative year, every 6 months for the subsequent 2 years, and annually thereafter when clinically indicated. BCC cases were reviewed every 6 months during the first two postoperative years and annually thereafter. At each visit, the operative site was examined by inspection and palpation. Cervical ultrasound was performed selectively in SCC at 3 and 6 months, then, when nodal involvement was suspected, median oncological follow‐up was 18 months (interquartile range 12–30 months). Suspicious clinical changes prompted biopsy, and recurrence was registered only on histological confirmation. Cases with fewer than 12 months of follow‐up were retained only if an oncological event had already occurred; those lost to follow‐up before 12 months without a recorded event were excluded at the selection stage rather than censored. The resulting median follow‐up is therefore conservative.

### 2.6. Outcome Measures

The primary outcome was histologically confirmed local recurrence of the index carcinoma, defined as carcinoma arising on the surgical scar or within 1 cm of the scar and judged clinically and histologically consistent with recurrence of the treated lesion. Scar irregularities and inflammatory changes were not counted as recurrence unless carcinoma was histologically confirmed. Second primary tumours—new carcinomas arising more than 1 cm from the scar or in a clearly distinct anatomical site—were recorded descriptively and excluded from the primary recurrence endpoint. This definition is consistent with the case‐attribution criteria described in Section 2.2 and was applied uniformly across all outcome assessments.

### 2.7. Statistical Approach

All analyses were descriptive. Categorical variables were summarised as counts and percentages. The study was designed to characterise real‐world postoperative management patterns and recurrence distributions under conventional excision conditions, rather than to estimate causal treatment effects. Two‐sided Fisher exact tests (FT) were used for predefined exploratory binary comparisons. *p*values are reported as exact descriptors of distributional imbalance under sparse‐event conditions; they were interpreted together with the underlying counts, biological plausibility and clinical context, not as stand‐alone evidence of treatment superiority, safety or equivalence. All FT codes, *p*values and 2 x 2 contingency tables are provided in Supporting Tables S-IV and S-V for full traceability. The limited number of endpoint events precluded multivariable analysis; the Fisher comparisons are exploratory, and subgroup findings are underpowered.

Comparisons between structured surveillance and early re‐excision in BCC were interpreted as descriptions of real‐world management pathways in a nonrandomised setting. Management allocation reflected clinical judgement influenced by anatomical subunit, margin category, depth, lesion size, patient age, comorbidity, histological risk and reconstructive feasibility. Given the low event count, emphasis was placed on recurrence distribution, effect direction and clinical coherence rather than isolated *p*value interpretation.

## 3. Results

### 3.1. Cohort Characteristics and Anatomical Distribution

The cohort comprised 238 surgically treated facial carcinoma cases: 172 BCC and 66 SCC. Both histotypes were concentrated in elderly age groups. Mean age was 74.6 years in BCC (median 76.0; range 31–103) and 78.2 years in SCC (median 77.5; range 43–102), with most patients in their eighth or ninth decade. Age distribution and related parameters are detailed in Supporting Table S-I and Supporting Figure S-1. Sex distribution is summarised in Table 1.

**TABLE 1:** Sex distribution by histotype.

Histotype	Sex	N	%
BCC	Male	90	52.3
	Female	82	47.7

SCC	Male	43	65.2
	Female	23	34.8

Anatomical distribution differed substantially between histotypes and informed the clinical interpretation of postoperative management. BCC was most frequently located on the nose (68 cases, 39.5%), followed by the ear (30 cases, 17.4%) and forehead (24 cases, 14.0%), whereas SCC was most frequent on the ear (23 cases, 34.8%), scalp (9 cases, 13.6%) and nose (8 cases, 12.1%). The corresponding anatomical distribution is shown in Supporting Figure S-2, and additional site‐specific considerations for margin interpretation are summarised in Supporting Table S-VI. This distribution is clinically relevant because nasal and periocular BCC often arises in subunits where functional and aesthetic constraints may limit the feasibility of wide re‐excision, whereas auricular and scalp SCC may present specific challenges at deep margins near cartilage, perichondrium or periosteum. Anatomical site was used for contextual interpretation and was not modelled as an independent causal variable.

Lesion size and depth characteristics are summarised in Table 2. BCC cases were more often smaller than 1 cm (63.4% vs 50.0% in SCC), consistent with earlier clinical presentation and slower growth. SCC cases showed a higher proportion of lesions between 1 and 2 cm. Nonclear margins were more frequent among larger lesions (≥ 1 cm: 54.2% vs < 1 cm: 40.8%; FT.3.6.3, *p* = 0.048) and among lesions with deep structural involvement (59.6% vs 42.9%; FT.3.6.2, *p* = 0.050), consistent with the known relationship between tumour extension and margin difficulty.

**TABLE 2:** Lesion size and depth by histotype.

Histotype	Size	N	%	Depth
BCC (*n* = 172)	< 1 cm	109	63.4	Superficial: 13 (7.6%)
	1–2 cm	49	28.5	Dermis: 124 (72.1%)
	> 2 cm	14	8.1	Deep str.: 35 (20.3%)

SCC (*n* = 66)	< 1 cm	33	50.0	Superficial: 16 (24.2%)
	1–2 cm	27	40.9	Dermis: 38 (57.6%)
	> 2 cm	6	9.1	Deep str.: 12 (18.2%)

*Note:* Deep structures include muscle, perichondrium or periosteum.

### 3.2. Anaesthetic and Reconstructive Management

Most procedures were performed under local anaesthesia (122/172, 70.9% in BCC; 55/66, 83.3% in SCC). Enhanced local or sedation‐assisted anaesthesia was used when dissection complexity required it. General anaesthesia was uncommon, reserved for larger lesions or patients with limited tolerance (12/172, 7.0% in BCC; 3/66, 4.5% in SCC). No anaesthesia‐related adverse events were recorded.

Reconstruction by histotype is shown in Table 3, and pooled reconstruction counts are provided in Supporting Table S-VII. Primary closure was used in 146/238 cases overall (61.3%). Local flap reconstruction was substantially more frequent in BCC (34.9% vs 13.6% in SCC), reflecting the predominance of BCC in contour‐critical nasal and periocular subunits. Skin grafting was proportionally more frequent in SCC (12.1% vs 4.1% in BCC; FT.3.4.1, *p* = 0.034), consistent with the distribution of SCC in auricular and scalp regions where local tissue mobility is limited. This difference in reconstructive pattern is clinically relevant because it reflects the distinct anatomical profiles of the two histotypes and may influence the feasibility of re‐excision. Perioperative morbidity was low overall (BCC: 19/172, 11.0%; SCC: 5/66, 7.6%).

**TABLE 3:** Reconstruction by histotype.

Reconstruction	BCC (*n* = 172)	SCC (*n* = 66)
Primary closure	99 (57.6%)	47 (71.2%)
Local flap	60 (34.9%)	9 (13.6%)
Skin graft^∗^	7 (4.1%)	8 (12.1%)
Flap + graft	3 (1.7%)	1 (1.5%)
Second intention	3 (1.7%)	1 (1.5%)

^∗^SCC vs BCC skin graft rate: *p* = 0.034 (Fisher exact test, FT.3.4.1).

### 3.3. Margin Status and Re‐Excision Decisions

In BCC, 90 cases (52.3%) achieved clear margins, 46 (26.7%) close margins and 36 (20.9%) positive margins. In SCC, 38 cases (57.6%) had clear margins, 13 (19.7%) close margins and 15 (22.7%) positive margins. Re‐excision rates by margin status are summarised in Table 4.

**TABLE 4:** Surgical re‐excision by histotype and margin status.

Histotype	Margin	Cases	Re‐excisions	Rate
BCC	Clear	90	0	0%
	Close	46	5	10.9%
	Positive	36	12	33.3%^†^

SCC	Clear	38	0	0%
	Close	13	1	7.7%
	Positive	15	5	33.3%^†^

^†^ *p* < 0.001 vs clear BCC (FT.3.3.8); *p* = 0.001 vs clear SCC (FT.3.3.9).

In both histotypes, early re‐excision was performed only after close or positive margins: zero clear‐margin cases in either histotype underwent re‐excision. Within BCC non‐clear‐margin cases, positive margins were more often followed by early re‐excision than close margins (33.3% vs 10.9%; FT.3.3.6, *p* = 0.026). The same directional pattern was observed in SCC (33.3% vs 7.7%), though small cell counts limited statistical discrimination (FT.3.3.7, *p* = 0.173). In SCC, larger lesions (> 2 cm) were more frequently re‐excised than smaller ones (50.0% vs 6.1% for < 1 cm; FT.3.3.3, *p* = 0.019), reflecting the combined influence of tumour size and oncological concern in this histotype.

Re‐excision decisions reflected clinical judgement rather than a uniform protocol as outlined in Section 2.7. Anatomical feasibility, depth of invasion, patient frailty and expected reconstructive burden all influenced whether re‐excision was pursued. Cases selected for surveillance were likely to differ from those selected for re‐excision in clinically relevant ways. This selection structure is essential for interpreting outcome comparisons between management pathways.

### 3.4. Oncological Outcomes—A) BCC

No endpoint recurrence was recorded after clear BCC margins (0/90). Endpoint recurrence according to histotype and margin status is summarised in Table 5. Among the 82 non‐clear‐margin BCC cases, six endpoint recurrences were identified (7.3%): 3/46 after close margins (6.5%) and 3/36 after positive margins (8.3%). Four additional recurrence‐like events, all in clear‐margin cases, were excluded from the primary endpoint because each arose more than 1 cm from the surgical scar in a clearly distinct anatomical site and was recorded as second primary disease rather than local recurrence of the index lesion, in line with the case‐attribution criteria defined in Section 2.2 (Supporting Table S-VIII).

**TABLE 5:** Endpoint recurrence by histotype and margin status.

Histotype	Margin	Cases	Recurrences	Rate
BCC	Clear	90	0	0.0%
	Close	46	3	6.5%
	Positive	36	3	8.3%

SCC	Clear	38	0	0.0%
	Close	13	0	0.0%
	Positive	15	3	20.0%^‡^

^‡^ *p* = 0.010 vs clear + close SCC margins (FT.3.5.2).

Among the 82 non‐clear‐margin BCC cases, 65 were initially managed by structured surveillance and 17 underwent early re‐excision (Table 6). Baseline clinical characteristics of these two non‐randomised management pathways are summarised descriptively in Supporting Table S-IX. Endpoint recurrence was recorded in 5/65 surveillance cases (7.7%) and in 1/17 re‐excision cases (5.9%). This result describes the observed recurrence distribution after clinically selected management pathways. It does not establish equivalence between surveillance and re‐excision, because allocation was nonrandom and reflected anatomical site, margin category, patient frailty, perceived oncologic risk, histological subtype when available, and expected reconstructive burden (FT.3.5.1, *p* = 1.000).

**TABLE 6:** Recurrence by management pathway in non‐clear‐margin BCC (*n* = 82).

Management pathway	Cases	Recurrences	Rate
Structured surveillance	65	5	7.7%
Early re‐excision	17	1	5.9%

*Note:* Allocation was nonrandom; this is a descriptive comparison only.

### 3.5. Oncological Outcomes—B) SCC

In SCC, all endpoint recurrences were confined to the positive‐margin subgroup: 3/15 positive‐margin cases recurred (20.0%), while no recurrence was recorded among clear‐margin (0/38) or close‐margin (0/13) SCC cases. The concentration of recurrences in the positive‐margin subgroup showed exploratory exact‐test support (FT.3.5.2, *p* = 0.010), but the clinical interpretation rests primarily on the convergence between this recurrence distribution, SCC biology and current postoperative risk concepts. No regional nodal or distant metastases were documented during the available follow‐up period.

The confinement of SCC recurrences to positive‐margin cases represents the most clinically relevant recurrence pattern observed in this cohort. Although based on only three endpoint events, this distribution is directionally coherent and clinically important because tumour at ink has a different postoperative meaning in SCC than in BCC.

### 3.6. Integrated Histotype‐Specific Outcome Pattern

The overall outcome pattern was structured rather than random in clinical appearance: clear margins were followed by no endpoint recurrence in either histotype; non‐clear‐margin BCC showed uncommon recurrence across selectively chosen postoperative pathways and SCC recurrence was confined to positive‐margin cases. These observations support the central premise of the study: postoperative margin status should be interpreted through histotype biology, anatomical feasibility, reconstructive burden and follow‐up reliability, rather than through margin distance alone.

## 4. A Histotype‐Specific Decision Framework

**FIGURE 1:**
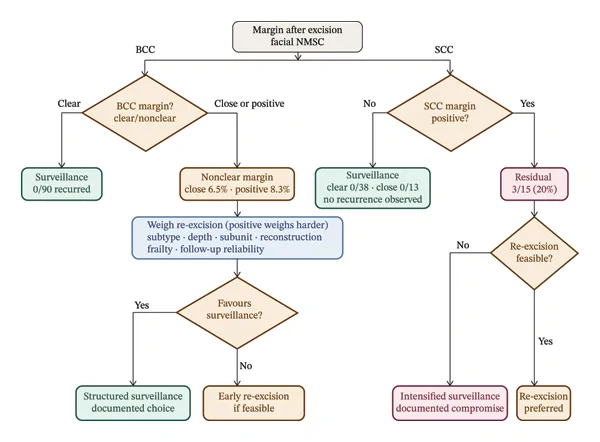
*Proposed histotype-specific decision framework for postoperative margin management of facial NMSC after conventional excision.* The critical margin threshold falls at a different point in each histotype. In BCC, a close margin already carries residual‐disease risk and is weighed alongside positive margins for possible re‐excision (clear vs nonclear threshold). In SCC, clear and close margins are managed by surveillance and only a positive margin drives re‐excision. Percentages and proportions shown in the boxes are the observed endpoint‐recurrence figures from the present cohort. The framework is an interpretive clinical‐reasoning aid, not a validated algorithm; every pathway requires individual clinical judgement.

Based on the descriptive findings of this cohort and on supporting literature, we propose a pragmatic framework for postoperative margin management of facial NMSC after conventional excision (Figure 1). The framework is intended as a structured clinical reasoning tool, not as a protocol derived from controlled comparative evidence. It organises the variables that influenced postoperative decisions in this cohort and that are clinically relevant to skin cancer care: histotype, margin category, anatomical and reconstructive context, patient‐level vulnerability and feasibility of reliable follow‐up. The contribution of the present cohort to this framework is limited and specific. The data support two empirical anchors: the absence of endpoint recurrence after clear margins in both histotypes, and the confinement of all SCC recurrences to the positive‐margin subgroup. These two observations are directly cohort‐derived, although both rest on small event numbers. The remaining structure of the framework, including the weighting of histological subtype, anatomical subunit, frailty and follow‐up reliability, and the lower re‐excision threshold proposed for SCC, derives predominantly from established literature and current guideline concepts rather than from statistically robust signals within this dataset. The framework should therefore be read as literature‐informed clinical reasoning calibrated against a small confirmatory cohort, not as a model validated by the present data.

For BCC with clear margins, routine surveillance remains appropriate. For BCC with nonclear margins, the decision requires case‐by‐case assessment across four dimensions: histological subtype risk; anatomical subunit and expected morbidity of re‐excision; patient frailty and comorbidity and follow‐up reliability. Structured surveillance can be considered when these conditions are favourable, provided it is understood as active monitoring with scheduled visits, careful scar examination, patient education and a low biopsy threshold, rather than passive observation.

For SCC, the decision threshold differs. Clear margins support surveillance according to clinical risk. Close‐margin SCC behaved like clear margins in this cohort, with no recurrence observed in this subgroup, and was likewise managed by surveillance rather than routine re‐excision. This surveillance should nonetheless remain risk‐informed, taking into account depth of invasion, differentiation, perineural invasion when reported, anatomical site, immunosuppression, frailty and feasibility of further surgery. Positive‐margin SCC should generally prompt timely re‐excision or an equivalent oncologic strategy whenever anatomically and medically feasible. Frailty or anatomical complexity may modify the operative plan, but they do not remove the oncologic relevance of tumour at ink. When re‐excision is not feasible or is declined, intensified surveillance should be documented as a risk‐adapted compromise, with the residual oncologic risk discussed explicitly with the patient.

## 5. Discussion

### 5.1. Histotype‐specific Interpretation of Postoperative Margins

The present findings support a histotype‐specific interpretation of nonclear margins after conventional excision, with a biological rationale consistent with existing literature. BCC grows by local extension, rarely metastasizes and residual microscopic disease after incomplete excision may not progress to clinically detectable recurrence within the relevant survival horizon, particularly in elderly patients [9, 13, 15, 18]. The 7%–8% recurrence rates observed in non‐clear‐margin BCC across both management pathways are therefore best interpreted as recurrence distributions within clinically selected pathways, not as proof that surveillance and re‐excision are interchangeable.

SCC behaves differently. The confinement of all SCC endpoint recurrences to the positive‐margin subgroup, against a background of zero recurrences after clear or close margins, is a descriptive finding with small absolute numbers but high clinical coherence. Residual SCC at the surgical margin carries biological risks that BCC generally does not: deeper tissue extension, perineural spread and, in a minority of cases, nodal involvement [4–6]. This difference supports a lower re‐excision threshold for SCC and supports treating close and positive SCC margins as clinically distinct categories. Close SCC margins may be considered for risk‐adapted surveillance in selected cases, whereas positive SCC margins generally warrant re‐excision or an equivalent oncologic strategy when feasible.

### 5.2. Structured Surveillance in Facial BCC

The comparable recurrence rates between surveillance and re‐excision pathways in non‐clear‐margin BCC (7.7% vs 5.9%) reflect clinical selection rather than therapeutic equivalence, as cases with greater oncological concern were preferentially re‐excised. Residual carcinoma was confirmed in only a minority of re‐excision specimens, consistent with the indolent behaviour of residual BCC and supporting selective rather than routine re‐excision in frail patients. These proportions are descriptive, given the retrospective derivation and small numbers.

Structured surveillance may therefore be clinically acceptable in appropriately selected BCC cases, under the conditions set out in Figure 1. Published cohorts support the observation that a substantial proportion of incompletely excised BCCs, particularly in elderly patients, do not recur within clinically relevant timeframes [19–21].

Structured surveillance should be considered an active management pathway. It requires scheduled clinical review, systematic scar assessment, prompt biopsy when changes are suspicious and patient education regarding warning signs. Without this infrastructure, observation should not be considered an adequate alternative to re‐excision.

### 5.3. Positive SCC Margins and Re‐Excision Threshold

The 20.0% recurrence rate after positive SCC margins in this cohort, against a background of zero recurrences after clear or close margins, represents the main SCC signal in the study. The absolute number of events is small (*n* = 3), but this does not make the observation clinically irrelevant. The strength of the finding lies in the convergence of recurrence localization, exploratory statistical support (*p* = 0.010), biological plausibility and current guideline‐consistent risk interpretation [6, 22, 23].

The clinical threshold for action in positive‐margin SCC should be lower than for BCC. Frailty and anatomical complexity are legitimate modifiers of the surgical approach—they may determine whether re‐excision is performed under local or general anaesthesia, whether it is combined with reconstruction or whether it is pursued at all. However, these factors should not reclassify positive SCC margins as an acceptable long‐term endpoint. When re‐excision is genuinely not feasible, the decision to observe must be documented as a deliberate, patient‐discussed, risk‐adapted compromise, with explicit intensification of the surveillance schedule.

### 5.4. The Role of Reconstruction in This Context

Reconstructive modality in this cohort reflected anatomical and operative complexity rather than functioning as an independent prognostic factor. Local flaps were more frequent in BCC, consistent with the predominance of nasal and periocular cases requiring contour‐preserving reconstruction. Skin grafting was more frequent in SCC, consistent with the distribution of SCC in auricular and scalp subunits with limited local tissue mobility.

Reconstructive complexity legitimately influences the re‐excision decision and provides contextual information that margin status alone cannot capture. Among the nine endpoint recurrences, reconstruction spanned the full operative range, from primary closure to combined flap and graft repair, with no concentration in any single modality. Reconstructive complexity therefore did not drive the recurrence pattern; rather, it acted upstream as one of the factors influencing the initial decision. This descriptive observation, based on small numbers, indicates only that recurrences were not confined to technically complex reconstructions.

### 5.5. Conventional Excision in Centres Without Routine Mohs Surgery

Mohs micrographic surgery remains the benchmark for margin‐controlled excision of selected high‐risk facial NMSC. The present study addresses a different and still common clinical setting: conventional excision in a centre where Mohs surgery was not routinely available. This distinction is essential for journal positioning. The manuscript does not argue that conventional excision is preferable to Mohs surgery for high‐risk facial tumours; it examines how postoperative margin information can be interpreted when conventional excision is the actual pathway used.

The recurrence profile of this cohort, namely zero recurrences after clear margins in either histotype, low recurrence rates in non‐clear‐margin BCC and recurrence confined to positive‐margin SCC, supports conventional excision as a clinically relevant pathway in centres without routine Mohs access, provided that case selection, surgical expertise, pathology quality and postoperative surveillance are maintained at a high standard. The proposed framework is intended to render that pathway explicit and reproducible, aligned with the biological differences between BCC and SCC.

### 5.6. Limitations

The retrospective single‐centre design and nonrandom treatment allocation are subject to confounding by anatomical site, margin category, depth, size, comorbidity, histotype and reconstructive feasibility. Endpoint recurrences were few, particularly in SCC (*n* = 3), limiting the precision of rate estimates. Margin topography (deep vs peripheral) was not consistently available for retrospective stratification. Median follow‐up was 18 months, which captures early recurrences but underestimates delayed events, particularly in indolent BCC subtypes recurring up to 3 to 5 years after treatment. The cohort was enriched for anatomically complex facial cases, limiting generalisability to low‐risk settings. Two further limitations are notable. First, the unit of analysis was the treated carcinoma rather than the patient: no unique patient identifier was available, and given the frequency of field cancerisation in facial NMSC, within‐patient clustering cannot be excluded and may modestly overstate the precision of the reported proportions. Second, SCC prognostic factors such as differentiation, perineural invasion, lymphovascular invasion and immunosuppression were not consistently recorded, so the SCC findings reflect margin status alone.

The proposed framework should therefore be interpreted as a retrospective clinical‐reasoning tool grounded in observed recurrence distributions, not a validated algorithm. Prospective validation with longer follow‐up, standardised margin‐topography reporting and functional outcome measures is required before broader recommendations can be made.

## 6. Conclusions

Postoperative management of nonclear margins after conventional facial excision of NMSC is best guided by histotype‐specific interpretation rather than by margin status alone.

Two findings are clinically relevant. In BCC, no recurrence occurred after clear margins, and recurrence remained uncommon in non‐clear‐margin cases across clinically selected management pathways. This pattern is compatible with the indolent biology of many residual BCCs and with structured surveillance in carefully selected patients. In SCC, all endpoint recurrences arose exclusively in the positive‐margin subgroup, with none recorded after clear or close margins. This finding, even with small absolute numbers, supports a lower threshold for re‐excision consideration in SCC than in BCC.

The proposed framework organises the clinical variables that govern the balance between oncologic clearance and functional preservation: histotype, margin category, anatomical subunit, reconstructive burden, patient frailty and follow‐up reliability. It should be used as an interpretive framework for postoperative decision‐making after conventional excision and should be prospectively validated with longer follow‐up, standardised margin‐topography reporting and patient‐centred functional outcomes.

## Author Contributions

F.P.: conceptualization, data curation, formal analysis, investigation, methodology, project administration, supervision, validation, visualization, writing–original draft, and writing–review and editing.

S.O.: conceptualization, data curation, formal analysis, investigation, supervision, validation, visualization, writing–original draft, and writing–review and editing.

G.D.: conceptualization, data curation, formal analysis, investigation, supervision, validation, visualization, writing–original draft, and writing–review and editing.

K.B.: conceptualization, data curation, formal analysis, investigation, validation, writing–original draft, and writing–review and editing.

D.S.: conceptualization, data curation, formal analysis, investigation, validation, writing–original draft, and writing–review and editing.

P.G.: conceptualization, data curation, formal analysis, investigation, validation, writing–original draft, and writing–review and editing.

C.R.: conceptualization, data curation, formal analysis, investigation, validation, writing–original draft, and writing–review and editing.

R.E.: conceptualization, data curation, formal analysis, investigation, validation, writing–original draft, and writing–review and editing.

M.B.: conceptualization, data curation, formal analysis, investigation, methodology, validation, visualization, writing–original draft, and writing–review and editing.

## Funding

No financial support was received for the research, authorship, or publication of this article.

Open access publishing facilitated by Universita degli Studi di Genova, as part of the Wiley ‐ CRUI‐CARE agreement.

## Ethics Statement

This retrospective study was conducted in accordance with institutional and national regulations. According to the policy of the Department of ENT and Stomatology, Centre Hospitalier des Escartons, Briancon, specific ethics committee approval was not required for retrospective analyses using fully anonymised data. Written informed consent for the use of anonymised clinical data was obtained from all patients where required by institutional procedures.

## Conflicts of Interest

The authors declare no conflicts of interest.

## Supporting Information

Additional supporting information can be found online in the Supporting Information section.

## Supporting information


**Supporting Information** Supporting Figure S‐1: Age distribution of treated cases. Supporting 2. Supporting Figure S‐2: Anatomical distribution of facial BCC and SCC cases. Supporting 3. Supporting Table S‐I: Age‐related parameters in BCC and SCC cases. Supporting 4. Supporting Table S‐II: Histologic subtypes of SCC and recurrence‐relevant features. Supporting 5. Supporting Table S‐III: Histologic subtypes of BCC and recurrence‐relevant features. Supporting 6. Supporting Table S‐IV: Register of exploratory two‐sided Fisher exact tests with p‐values. Supporting 7. Supporting Table S‐V: Exact 2 x 2 contingency tables for all Fisher exact tests. Supporting 8. Supporting Table S‐VI: Anatomical site, margin interpretation and recurrence‐relevant surgical context. Supporting 9. Supporting Table S‐VII: Pooled distribution of reconstruction techniques (*n* = 238). Supporting 10. Supporting Table S‐VIII: Case‐level summary of endpoint local recurrences and second primary tumours excluded from the primary recurrence endpoint. Supporting 11. Supporting Table S‐IX: Descriptive clinical context of structured surveillance and early re‐excision pathways among non‐clear‐margin BCC cases.

## Data Availability

All relevant data are reported in the main text and supporting tables. Additional materials may be obtained from the corresponding author upon reasonable request.
