# Supplementary material for: Postoperative Margin Management After Conventional Excision of Facial Basal and Squamous Cell Carcinomas: A Histotype‐Specific Framework
Source: J Skin Cancer. 2026 Aug 28;2026:9952281. doi: 10.1155/jskc/9952281 (PMC13522925; doi:10.1155/jskc/9952281)
Supplement: Supplementary file 1 [file JSKC-2026-9952281-s001.zip › Supporting_FINAL-Perottino_v7.docx]

**Supporting Material**

**Postoperative Margin Management After Conventional Excision of Facial Basal and Squamous Cell Carcinomas: A Histotype-Specific Framework**

Flavio Perottino, Stefano Ottoboni, Giulia Dalmasso, Katia Barbaro, Daniele Sagrafoli, Paola Ghisellini, Cristina Rando, Roberto Eggenhöffner and Mario Borgione

# Supporting note on treated cases and endpoint attribution

All denominators in the Supporting tables refer to surgically treated facial carcinoma cases, unless explicitly stated otherwise. Local recurrence denotes histologically confirmed carcinoma arising at the scar, or within 1 cm of the scar, and judged consistent with recurrence of the treated lesion. Clinically distinct new carcinomas arising outside this area were recorded as second primary tumours and were not counted as endpoint recurrences.

The Supporting tables support traceability of the descriptive analyses. Fisher exact tests are reported as predefined exploratory checks of distributional imbalance under sparse-event conditions. They should be interpreted together with the underlying counts, recurrence localization, biological plausibility, and clinical context. They are not intended to establish treatment equivalence, causal effects of reconstruction or management pathway, or independent prognostic models.

# Supporting Figure S-1. Age distribution of treated cases

The age distribution shows a predominance of older cases, particularly between 67 and 86 years, with a peak in the 77-81-year interval and cases also represented above 90 years. This demographic profile supports the clinical relevance of patient vulnerability, surgical burden, and feasibility of structured follow-up when interpreting postoperative management after conventional excision. The figure provides demographic context only.

# Supporting Table S-I. Age-related parameters in BCC and SCC cases

| **Parameter** | **Subcategory** | **BCC** | **SCC** |
| --- | --- | --- | --- |
| Cases |  | 172 | 66 |
| Mean age |  | 74.63 | 78.21 |
| Median age |  | 76.00 | 77.50 |
| Minimum |  | 31 | 43 |
| Maximum |  | 103 | 102 |
| Percentile | 25th | 68.00 | 72.00 |
| Percentile | 50th | 76.00 | 77.50 |
| Percentile | 75th | 83.00 | 86.25 |

Both histotypes were concentrated in elderly age groups, with SCC cases showing a slightly higher mean age than BCC cases. These data define the geriatric context of the cohort and support interpretation of re-excision decisions in relation to comorbidity, surgical tolerance, and reconstructive burden.

# Supporting Figure S-2. Anatomical distribution of facial BCC and SCC cases

BCC and SCC showed distinct anatomical distributions. BCC was predominantly located on the nose, followed by the ear and forehead, whereas SCC was more frequent on the auricle and scalp. This distribution provides anatomical context for postoperative margin interpretation, because nasal and periocular BCCs frequently arise in functionally and aesthetically constrained subunits, while auricular and scalp SCCs may involve deep planes close to cartilage, perichondrium, or periosteum. The figure is descriptive and was not used to infer site-specific recurrence risk.

# Supporting Table S-II. Histologic subtypes of squamous cell carcinoma and recurrence-relevant features

| **Subtype/pattern** | **Risk level used for contextual interpretation** | **Common features** | **Recurrence-relevant interpretation** |
| --- | --- | --- | --- |
| Keratinizing | Moderate | Well or moderately differentiated squamous phenotype; often linked to chronic actinic damage | Variable; interpreted with grade, depth, and margin status |
| Keratoacanthoma-like | Usually low-to-intermediate, context dependent | Crateriform architecture; may overlap clinically with rapidly growing lesions | Risk interpretation depends on invasion and differentiation |
| Sarcomatoid/spindle-cell | High | Spindle-cell morphology; may show infiltrative growth | Potentially aggressive; requires cautious margin interpretation |
| Adenoid/acantholytic | High | Pseudoglandular or acantholytic architecture | Higher-risk morphology in many classification systems |
| Adenosquamous | Very high | Squamous carcinoma with glandular differentiation | Clinically aggressive in many reported series |
| Desmoplastic | High | Dense stromal reaction; perineural or perivascular invasion may be present | Associated with infiltrative local behaviour |
| Clear cell | Uncertain/moderate | Clear cytoplasm; diagnostic distinction may be required | Insufficient data for independent inference in this cohort |
| Pigmented | Uncertain | Melanin-containing lesion that may mimic melanoma clinically or histologically | Primarily relevant for diagnosis rather than outcome inference here |
| Follicular | Uncertain/high depending on invasion | Follicular differentiation or origin; may mimic adnexal or metastatic patterns | Not analysed as an independent recurrence predictor in this dataset |

This table summarises histological patterns relevant to SCC risk interpretation. The categories are provided as contextual pathology descriptors and literature-informed clinical considerations. Subtype-level recurrence analysis was not performed because endpoint events were few and retrospective pathology descriptors were not sufficiently uniform for robust stratification; the table should therefore not be read as an internally validated prognostic model.

# Supporting Table S-III. Histologic subtypes of basal cell carcinoma and recurrence-relevant features

| **Subtype** | **Risk level used for contextual interpretation** | **Descriptive features** | **Recurrence-relevant interpretation** |
| --- | --- | --- | --- |
| Nodular | Low | Well-defined nodules; usually limited stromal reaction | Generally low recurrence risk when completely excised |
| Superficial | Low | Predominantly horizontal extension with superficial epidermal attachment | Low recurrence risk in appropriate clinical contexts |
| Micronodular | High | Small invasive nests with possible tentacle-like spread and deep involvement | Greater risk of subclinical extension and incomplete clearance |
| Infiltrative | High | Irregular margins and desmoplastic stromal response; perineural invasion may occur | Higher local recurrence risk; margin interpretation requires caution |
| Sclerodermiform/  morpheaform | High | Dense stroma trapping thin tumour strands; difficult gross assessment | High risk of occult extension and positive/close margins |
| Infundibulocystic | Low-to-moderate | Basaloid cells with follicular-type differentiation | Recurrence relevance uncertain and context dependent |
| Fibroepithelial | Low | Anastomosing basaloid strands with fibrovascular stroma | Generally low recurrence risk |
| Basosquamous | Very high | Mixed basal and squamous differentiation | Requires cautious management because of more aggressive behaviour |

This table provides histological context for interpreting the BCC cohort. The categories are used to support clinical interpretation of margin status and management selection. They were not analysed as independent predictors of recurrence. Management decisions were interpreted as the combined result of histotype, margin status, anatomical constraints, patient vulnerability, reconstructive feasibility, and follow-up reliability rather than as a function of subtype alone.

# Supporting Table S-IV. Register of exploratory two-sided Fisher exact tests

The tests listed below document the exact binary comparisons used for traceability. P-values are reported as exploratory descriptors of distributional imbalance under sparse-event conditions. They should be interpreted together with the corresponding counts and the clinical structure of the cohort. A non-significant p-value does not demonstrate equivalence between management pathways, and a significant or borderline p-value does not establish causality.

| **Code** | **Exploratory comparison** | **Data used** | **Two-sided Fisher p** | **Descriptive interpretation** |
| --- | --- | --- | --- | --- |
| FT.3.3.1 | Histotype vs lesion size <1 cm | BCC: 109/172 (63.4%) vs SCC: 33/66 (50.0%) | 0.076 | BCC cases were more often <1 cm, with limited exact-test support. |
| FT.3.3.2 | BCC recurrence: >2 cm vs <1 cm | >2 cm: 2/14 (14.3%) vs <1 cm: 1/109 (0.9%) | 0.034 | Endpoint recurrence was more frequent in larger BCCs, with small absolute counts. |
| FT.3.3.3 | SCC early re-excision: >2 cm vs <1 cm | >2 cm: 3/6 (50.0%) vs <1 cm: 2/33 (6.1%) | 0.019 | Larger SCCs were more often managed with early re-excision. |
| FT.3.3.4 | SCC early re-excision: deep structures vs superficial | Deep: 2/12 (16.7%) vs superficial: 1/16 (6.3%) | 0.560 | A numerical difference was present but cell counts were very small. |
| FT.3.3.5 | BCC early re-excision: deep structures vs superficial | Deep: 5/35 (14.3%) vs superficial: 0/13 (0.0%) | 0.304 | The direction was compatible with escalation according to depth, but uncertainty remained substantial. |
| FT.3.3.6 | BCC early re-excision: positive vs close margins | Positive: 12/36 (33.3%) vs close: 5/46 (10.9%) | 0.026 | Positive margins were more often followed by early re-excision than close margins. |
| FT.3.3.7 | SCC early re-excision: positive vs close margins | Positive: 5/15 (33.3%) vs close: 1/13 (7.7%) | 0.173 | The same direction was observed in SCC, but sparse counts limited discrimination. |
| FT.3.3.8 | BCC early re-excision: positive vs clear margins | Positive: 12/36 (33.3%) vs clear: 0/90 (0.0%) | 6.43 ×10^-8^ | Early re-excision was concentrated in positive-margin BCC compared with clear-margin BCC. |
| FT.3.3.9 | SCC early re-excision: positive vs clear margins | Positive: 5/15 (33.3%) vs clear: 0/38 (0.0%) | 0.001 | Early re-excision was concentrated in positive-margin SCC compared with clear-margin SCC. |
| FT.3.4.1 | Histotype vs reconstruction: skin graft vs all other modalities | BCC: 7/172 (4.1%) vs SCC: 8/66 (12.1%) | 0.034 | Skin grafting was proportionally more frequent in SCC than BCC. |
| FT.3.4.2 | BCC reconstruction vs second intervention after initial surveillance | Local flap: 6/55 (10.9%) vs primary closure: 14/90 (15.6%) | 0.470 | No clear difference was evident under sparse-event conditions. |
| FT.3.4.3 | SCC reconstruction vs second intervention after initial surveillance | Skin graft: 1/6 (16.7%) vs local flap: 1/9 (11.1%) | 1.000 | The comparison has very limited discrimination because of extremely small counts. |
| FT.3.5.1 | BCC recurrence: surveillance vs early re-excision among non-clear margins | Surveillance: 5/65 (7.7%) vs early re-excision: 1/17 (5.9%) | 1.000 | Observed proportions were similar, but the comparison was descriptive and non-randomised. |
| FT.3.5.2 | SCC recurrence: positive vs non-positive margins | Positive: 3/15 (20.0%) vs non-positive: 0/51 (0.0%) | 0.010 | Endpoint recurrences were confined to positive-margin SCC cases in this cohort. |
| FT.3.6.1 | Site complexity vs flap-based repair | Complex sites: 63/155 (40.6%) vs other sites: 10/83 (12.0%) | 3.43 ×10^-6^ | Flap-based repair was more frequent in anatomically constrained sites. |
| FT.3.6.2 | Depth vs non-clear margins | Deep structures: 28/47 (59.6%) vs non-deep: 82/191 (42.9%) | 0.050 | Close or positive margins were more frequent with deep extension, with borderline exact evidence. |
| FT.3.6.3 | Size vs non-clear margins | ≥1 cm: 52/96 (54.2%) vs <1 cm: 58/142 (40.8%) | 0.048 | Close or positive margins were more frequent in lesions ≥1 cm. |

# Supporting Table S-V. Exact 2 x 2 contingency tables supporting Supporting Table S-IV

| **Code** | **Comparison** | **Group A** | **Event yes** | **Event no** | **Total** | **Group B** | **Event yes** | **Event no** | **Total** |
| --- | --- | --- | --- | --- | --- | --- | --- | --- | --- |
| FT.3.3.1 | Histotype vs <1 cm size | BCC | 109 | 63 | 172 | SCC | 33 | 33 | 66 |
| FT.3.3.2 | BCC recurrence: >2 cm vs <1 cm | >2 cm | 2 | 12 | 14 | <1 cm | 1 | 108 | 109 |
| FT.3.3.3 | SCC early re-excision: >2 cm vs <1 cm | >2 cm | 3 | 3 | 6 | <1 cm | 2 | 31 | 33 |
| FT.3.3.4 | SCC early re-excision: deep vs superficial | Deep structures | 2 | 10 | 12 | Superficial | 1 | 15 | 16 |
| FT.3.3.5 | BCC early re-excision: deep vs superficial | Deep structures | 5 | 30 | 35 | Superficial | 0 | 13 | 13 |
| FT.3.3.6 | BCC early re-excision: positive vs close | Positive margin | 12 | 24 | 36 | Close margin | 5 | 41 | 46 |
| FT.3.3.7 | SCC early re-excision: positive vs close | Positive margin | 5 | 10 | 15 | Close margin | 1 | 12 | 13 |
| FT.3.3.8 | BCC early re-excision: positive vs clear | Positive margin | 12 | 24 | 36 | Clear margin | 0 | 90 | 90 |
| FT.3.3.9 | SCC early re-excision: positive vs clear | Positive margin | 5 | 10 | 15 | Clear margin | 0 | 38 | 38 |
| FT.3.4.1 | Histotype vs skin grafting | BCC | 7 | 165 | 172 | SCC | 8 | 58 | 66 |
| FT.3.4.2 | BCC reconstruction vs second intervention after surveillance | Local flap | 6 | 49 | 55 | Primary closure | 14 | 76 | 90 |
| FT.3.4.3 | SCC reconstruction vs second intervention after surveillance | Skin graft | 1 | 5 | 6 | Local flap | 1 | 8 | 9 |
| FT.3.5.1 | BCC recurrence: surveillance vs early re-excision | Surveillance | 5 | 60 | 65 | Early re-excision | 1 | 16 | 17 |
| FT.3.5.2 | SCC recurrence: positive vs non-positive margins | Positive margin | 3 | 12 | 15 | Non-positive margin | 0 | 51 | 51 |
| FT.3.6.1 | Site complexity vs flap-based repair | Complex sites | 63 | 92 | 155 | Other sites | 10 | 73 | 83 |
| FT.3.6.2 | Depth vs non-clear margins | Deep structures | 28 | 19 | 47 | Non-deep | 82 | 109 | 191 |
| FT.3.6.3 | Size vs non-clear margins | ≥1 cm | 52 | 44 | 96 | <1 cm | 58 | 84 | 142 |

For FT.3.4.2 and FT.3.4.3, the event is second intervention after initial structured surveillance. For FT.3.6.1, the event is flap-based repair. For FT.3.6.2 and FT.3.6.3, the event is a non-clear margin, defined as close or positive. For FT.3.5.2, the event is endpoint local recurrence.

# Supporting Table S-VI. Anatomical site, margin interpretation, and recurrence-relevant surgical context

| **Anatomical site** | **Typical tumour pattern in this cohort context** | **Common margin pattern** | **Clinical interpretation** |
| --- | --- | --- | --- |
| Nose | Predominantly BCC | Often close | Tissue-sparing surgery is frequent because of nasal contour and airway/aesthetic constraints; interpretation requires integration with subtype and margin category. |
| Ear/auricular region | SCC and BCC | Close or positive may occur | Cartilage and perichondrial planes can constrain deep clearance; SCC in this site requires particular caution. |
| Periocular region/inner canthus | Predominantly BCC | Close or positive may occur | Conservative margins may be selected to preserve eyelid competence and ocular function. |
| Cheek | Predominantly BCC | Often clear | Greater tissue availability may allow more generous excision and simpler closure. |
| Scalp | Predominantly SCC in this cohort context | Clear or positive | Chronic actinic damage and deep plane involvement may increase management complexity. |
| Forehead | Predominantly BCC | Clear or close | Intermediate surgical complexity depending on lesion size, depth, and reconstruction requirements. |

This table provides anatomical context for postoperative interpretation. Anatomical site was considered jointly with histotype, size, depth, margin status, patient vulnerability, reconstructive feasibility, and follow-up reliability. It was not analysed as an independent prognostic model, because event counts and retrospective documentation did not support reliable site-specific recurrence modelling.

# Supporting Table S-VII. Pooled distribution of reconstruction techniques

| **Technique** | **No. of cases** | **Percentage** |
| --- | --- | --- |
| Primary closure | 146 | 61.3% |
| Local flap | 69 | 29.0% |
| Skin graft | 15 | 6.3% |
| Flap plus graft | 4 | 1.7% |
| Secondary intention | 4 | 1.7% |
| Total | 238 | 100.0% |

The pooled reconstruction counts are derived from the histotype-stratified reconstruction table in the main manuscript. The distribution supports the interpretation that closure strategy reflects defect geometry, anatomical site, surgical feasibility, and reconstructive burden. Reconstruction type should therefore be read as contextual information for postoperative decision-making, not as an isolated oncological risk factor.

**Supporting Table S-VIII**

Supporting Table S-VIII provides case-level traceability of the recurrence endpoint. It lists every histologically confirmed endpoint local recurrence in the cohort, with histotype, anatomical site, lesion size, depth, margin category, primary reconstruction, initial post-excision pathway, and histological confirmation. All endpoint recurrences arose in non-clear-margin cases, and all squamous cell carcinoma recurrences were confined to the positive-margin subgroup. Four clinically distinct carcinomas arising in clear-margin basal cell carcinoma cases were classified as second primary tumours under the predefined attribution criteria and were excluded from the endpoint; they are listed separately below the main table. The table supports traceability only and should not be read as a subtype-level prognostic model, because endpoint events were few and management allocation was non-random.

**Supporting Table S-VIII.** *Case-level summary of endpoint local recurrences (n = 9). Depth “Deep” denotes invasion of muscle, perichondrium, or periosteum.*

| Case ID | Histotype | Site | Size | Depth | Margin | Primary reconstruction | Initial post-excision pathway | Histologic confirmation |
| --- | --- | --- | --- | --- | --- | --- | --- | --- |
| B-69 | BCC | Ear | 1-2 cm | Dermis | Positive | Primary closure | Structured surveillance | Yes |
| B-74 | BCC | Nose | 1-2 cm | Dermis | Positive | Second intention | Structured surveillance | Yes |
| B-75 | BCC | Nose | >2 cm | Dermis | Close | Flap + graft | Structured surveillance | Yes |
| B-76 | BCC | Nose | >2 cm | Dermis | Close | Local flap | Structured surveillance | Yes |
| B-107 | BCC | Nose | <1 cm | Deep | Close | Primary closure | Structured surveillance | Yes |
| B-193 | BCC | Nose | 1-2 cm | Deep | Positive | Local flap | Early re-excision | Yes |
| S-43 | SCC | Nasolabial fold | 1-2 cm | Deep | Positive | Primary closure | Structured surveillance | Yes |
| S-46 | SCC | Scalp | >2 cm | Dermis | Positive | Flap + graft | Early re-excision | Yes |
| S-234 | SCC | Ear | >2 cm | Dermis | Positive | Primary closure | Early re-excision | Yes |

Recurrence-like events excluded from the endpoint (second primary tumours).

Four clinically distinct carcinomas arising in clear-margin BCC cases were classified as second primary tumours under the predefined attribution criteria (carcinoma arising more than 1 cm from the surgical scar or in a clearly distinct anatomical subunit) and were excluded from the local-recurrence endpoint.

| Case ID | Site | Size | Depth |
| --- | --- | --- | --- |
| B-68 | Forehead | 1-2 cm | Dermis |
| B-133 | Nose | <1 cm | Dermis |
| B-139 | Nose | <1 cm | Dermis |
| B-161 | Ear | >2 cm | Deep |

*Abbreviations: BCC, basal cell carcinoma; SCC, squamous cell carcinoma.*

**Supporting Table S-IX**

Supporting Table S-IX summarises the baseline clinical context of the two management pathways among non-clear-margin basal cell carcinoma cases. It reports age, sex, anatomical site grouping, lesion size, depth, margin category, reconstruction, and AIOM risk category for cases managed by structured surveillance versus early re-excision. The table is presented purely descriptively to make the non-random allocation of management explicit; no statistical comparison was performed. It is not a causal balance table and does not support inference of any treatment effect. The observed differences, in particular the higher proportion of positive margins among re-excised cases, reflect the clinical reasoning that guided pathway selection rather than any measured effect of one pathway over the other.

**Supporting Table S-IX.** *Baseline context of management pathways in non-clear-margin BCC (n = 82). Descriptive comparison only; allocation was non-random and no significance testing was applied.*

| Characteristic | Structured surveillance (n=65) | Early re-excision (n=17) |
| --- | --- | --- |
| Median age, years (range) | 77 (40-92) | 75 (41-90) |
| Male sex | 33 (50.8%) | 12 (70.6%) |
| Anatomically constrained site* | 49 (75.4%) | 14 (82.4%) |
| Lesion size <1 cm | 40 (61.5%) | 9 (52.9%) |
| Lesion size 1-2 cm | 19 (29.2%) | 7 (41.2%) |
| Lesion size >2 cm | 6 (9.2%) | 1 (5.9%) |
| Deep invasion** | 16 (24.6%) | 5 (29.4%) |
| Close margin | 41 (63.1%) | 5 (29.4%) |
| Positive margin | 24 (36.9%) | 12 (70.6%) |
| Flap-based reconstruction | 25 (38.5%) | 7 (41.2%) |
| High-risk (AIOM) | 52 (80.0%) | 14 (82.4%) |

**Anatomically constrained sites: nose, eyelid/periocular, ear, nasolabial fold. **Deep invasion: muscle, perichondrium, or periosteum. AIOM, Associazione Italiana di Oncologia Medica risk classification.*
